# Photoelectrochemical detection of alpha-fetoprotein based on ZnO inverse opals structure electrodes modified by Ag_2_S nanoparticles

**DOI:** 10.1038/srep38400

**Published:** 2016-12-06

**Authors:** Yandong Jiang, Dali Liu, Yudan Yang, Ru Xu, Tianxiang Zhang, Kuang Sheng, Hongwei Song

**Affiliations:** 1 State Key Laboratory on Integrated Optoelectronics, College of Electronic Science and Engineering, Jilin University, 2699 Qianjin Street, Changchun 130012, China; 2 China-Japan Union Hospital of Jilin University, Changchun 130012, China

## Abstract

In this work, a new photoelectrochemical biosensor based on Ag_2_S nanoparticles (NPs) modified macroporous ZnO inverse opals structure (IOs) was developed for sensitive and rapid detection of alpha fetal protein (AFP). Small size and uniformly dispersed Ag_2_S NPs were prepared using the Successive Ionic Layer Adsorption And Reaction (SILAR) method, which were adsorbed on ZnO IOs surface and frame work as matrix for immobilization of AFP. The composite structure of ZnO/Ag_2_S expanded the scope of light absorption to long wavelength, which can make full use of the light energy. Meanwhile, an effective matching of energy levels between the conduction bands of Ag_2_S and ZnO are beneficial to the photo-generated electrons transfer. The biosensors based on FTO (fluorine-doped tinoxide) ZnO/Ag_2_S electrode showed enough sensitivity and a wide linear range from 0.05 ng/mL to 200 ng/mL with a low detection limit of 8 pg/mL for the detection of AFP. It also exhibited high reproducibility, specificity and stability. The proposed method was potentially attractive for achieving excellent photoelectrochemical biosensor for detection of other proteins.

Primary liver cancer is known as malignant tumor, which is a serious threat to health and has a high mortality rate in the world^1^,^2^. Fast and accurate early detection of cancer biomarker is vital for clinical diagnosis^3^, thus, specific biomarkers are highly needed^4^. AFP is an oncogenic glycoprotein which is normally expressed during gestation and originally identified in the human fetus in 1956^5^, but an elevated AFP concentration in adult plasma may be an early symptom of malignant tumor. AFP can act as the most important biomarkers in the diagnosis and targeting of liver cancer.

In the past few years, many efforts had been made to detect AFP, such as enzyme-linked immunosorbent assay^6^,^7^, electrochemiluminescence^8^, fluorescence biosensor^9^, surface plasmon resonance immunoassays^10^ and electrochemical immunoassay^11^,^12^. Although some results were obtained, sophisticated instruments, significant sample volume, limited sensitivity, and long detection time limited the clinical application^13^. To develop the clinical detection, a novel, highly sensitive and alternative detection method of AFP is desired.

Due to simple structure and easily operation allowing rapid, high-throughput biological assay, PEC immunosensors were widely used in the analytical methods^14^,^15^. Immunochemical reactions at an electrode surface alter photocurrent generation and thus provide information about the respective biological process. Conventional immunoassays require antibody or antigen labelling with biomarkers for signal generation^16^. Enzyme-based PEC biosensors display high sensitivity, selectivity, simplicity, low cost, and minimal sample consumption^17^,^18^, but the process of introducing the enzyme is complicated and enzyme inherent instability at the same time makes it easy inactivation in the external environment^16^. Therefore, to develop non-enzymatic biosensor with high sensitivity, stability and selectivity is the requirement of the science and technology^19^. The prominent advantage of PEC-based non-enzymatic biosensors is the possibility of utilizing photo-holes in the VB of a semiconductor to facilitate oxidation of chemical and/or biological components in a liquid or gas phase^20^. Compared with enzyme-based PEC biosensing, non-enzymatic PEC detection is a more promising method, which need not the sample to be labeled and has higher stability and durability against the external environment. Considerable effort has been invested in developing non-enzymatic PEC biosensors^15^,^21^.

ZnO is one of the most extensively studied semiconductor oxides due to remarkable physical and chemical properties, presenting the most promising candidate in different applications, such as photocatalysis^22^,^23^, solar cells^24^,^25^, PEC water splitting^26^,^27^, and sensing applications^28^,^29^. Furthermore, its excellent thermal, chemical, low density, good biological compatibility and excellent photochemical stability^30^,^31^ make it attractive in PEC bioanalysis^32^,^33^. Many ZnO nanostructures, such as nanorods^29^, nanotubes^34^, nanowires^11^, nanosheets^35^ and nanoflowers^36^, have been applied in biosensor. Despite of this, the usage of ZnO without modification in PEC-based bioanalysis has some limitations because of its inherent wide band gap which results in a strong absorption in the UV region. It is noting that most biomolecules are very unstable under UV irradiation, the high activity of photo-holes produced in the VB of ZnO upon light illumination is disadvantageous to the biological molecules^37^. The current problem for ZnO electrode is the efficient utilization of the visible light^38^. As an important narrow band gap semiconductor material, Ag_2_S has a large absorption coefficient and a direct band gap of Eg~1.1 eV^39^, which has been successfully used for photocatalysis^40^ and photovoltaic cells^41^,^42^. Besides, Ag_2_S possesses an ultralow solubility product constant (Ksp = 6.3 × 10^−50^), which guarantees that the least amount of Ag^+^ ion is released into the biological surroundings, Ag_2_S possesses negligible toxicity compared to other commonly used narrow band gap materials^23^,^43^,^44^, which is advantageous to bioanalysis. So far, little work was carried out to use Ag_2_S NPs in PEC biosensor. It is a new method to composite ZnO with Ag_2_S to improve visible absorption and promote the effective separation of photo-generated charges.

In this work, we report on the synthesis of Ag_2_S NPs modified ZnO IOs photoelectrode used for immunosensor of AFP. The immunosensor with enhanced photocurrent intensity and less electron-hole recombination is desirable. As a 3D macropore structure, IOs possess a large surface area, which is advantageous to the electronic transmission and biomolecule immobilization^33^. Coupling of Ag_2_S with ZnO IOs could facilitate charges separation due to the quick electron transfer from the conduction band of the small band gap semiconductor to the conduction band of the large one^45^. Our results showed that the photocurrent of the composite electrodes was significantly enhanced due to the formation of ZnO/Ag_2_S composite electrodes. The electrodes also demonstrated good sensitivity and repeatability.

## Results and Discussion

### Characterizations of the FTO/ZnO/Ag_2_S composited electrode

**Figure 1:**
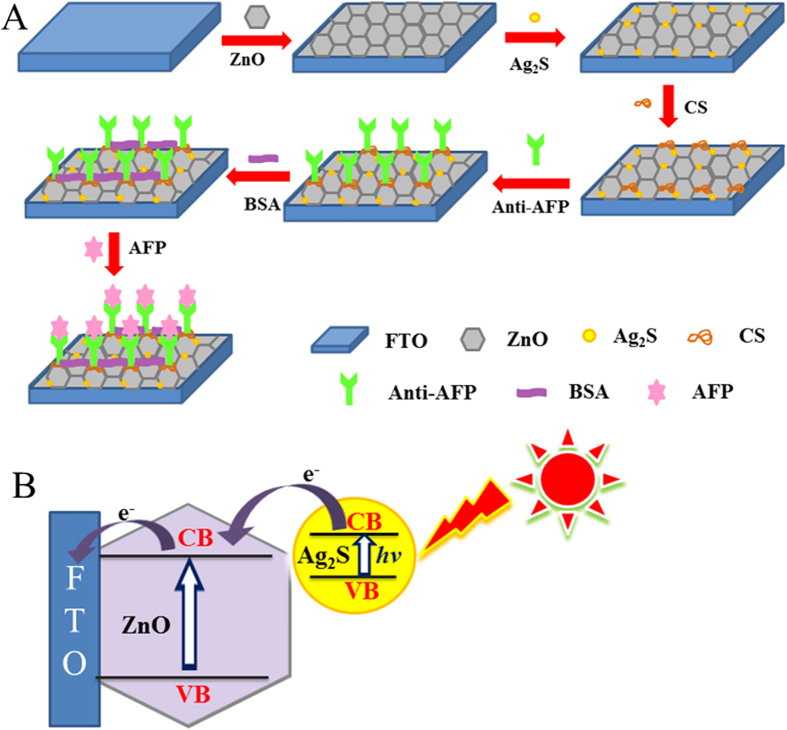
(**A**) The fabrication procedure of the immunosensor. (**B**) The photocurrent generation principle of ZnO/Ag_2_S modified electrode.

Figure 1A shows the fabrication procedure of the immunosensor. ZnO/Ag_2_S hybrid modified electrodes were obtained by successive Ag^+^ and S^2−^ adsorption on ZnO IOs electrodes, which combines the excellent charge transport property with absorption property of the ZnO/Ag_2_S^46^. As a biocompatible material with high permeability, CS was fixed on FTO/ZnO/Ag_2_S electrode for further immobilization of Anti-AFP antibody (Ab), then AFP was detected based on the specificity binding of antigen-antibody. Figure 1B shows photocurrent generation principle of ZnO/Ag_2_S modified electrodes. Due to matching of energy levels between ZnO and Ag_2_S, The loading of Ag_2_S NPs can lead to more efficient light absorption and consequently increase the photocurrent response by more electron injection from the excited Ag_2_S to the conduction band of ZnO.

**Figure 2:**
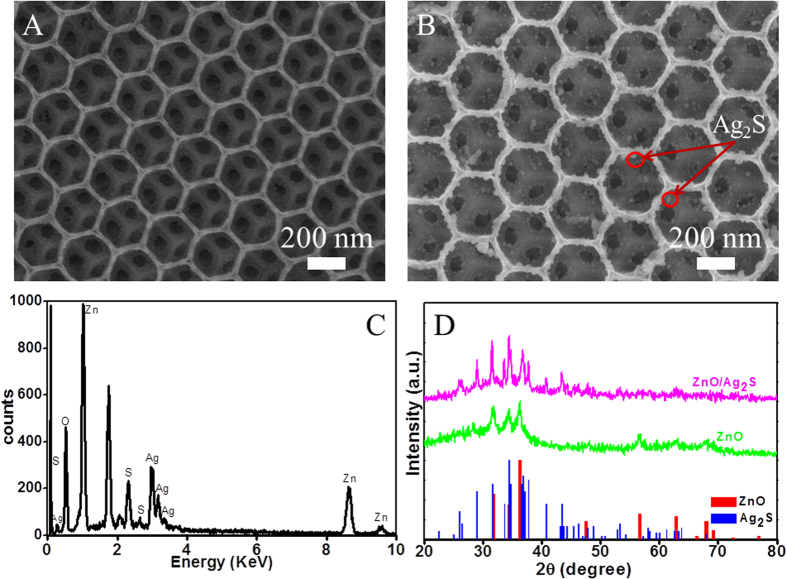
SEM image of ZnO IOs (**A**), ZnO modified with Ag_2_S NPs (**B**), (**C**) EDX analysis of Ag_2_S modified ZnO IOs of 3 SILAR cycle and (**D**) XRD patterns of ZnO IOs and ZnO/Ag_2_S composited electrode.

ZnO IOs were fabricated by the sol-gel method according to our previous report with a slight modification (See in Supplementary information). Figure 2A shows the field emission scanning electron microscope (SEM) image of the surface morphology and microstructure of the synthesized ZnO IOs. ZnO IOs display an ordered pore structure of three dimensional space, with lattice constant of ~251 nm. Figure 2B shows that some Ag_2_S NPs were deposited on frame work and outer surfaces after 3 SILAR cycles, and the average size of the Ag_2_S NPs was about 15 nm. This indicates that the Ag- and S^2−^ ions were easily diffused into the pores of the IOs without obvious aggregation and pore clogging to form Ag_2_S nanocrystallites. In order to further verify compound ZnO/Ag_2_S, the corresponding energy dispersive x-ray (EDX) spectrum of the FTO/ZnO/Ag_2_S was carried out, as shown in Fig. 2C. The observed peaks for Zn, O, Ag and S further confirmed that the substance was composed of ZnO/Ag_2_S. In order to investigate the structure of the ZnO/Ag_2_S, the crystalline phases of the ZnO and ZnO/Ag_2_S were characterized by XRD, as shown in Fig. 2D. The XRD pattern of ZnO showed good hexagonal matching (JCPDS, card no. 36-1451), no peaks of impurity were observed, indicating that the ZnO IOs sample was pure in hexagonal phase. After Ag_2_S deposition, peaks corresponding to α-Ag_2_S (JCPDS card no. 14-0072) were observed, further indicating the formation of ZnO IOs/Ag_2_S NPs composites.

### Optical and photoelectrochemical properties of the Ag_2_S NPs modified ZnO IOs

**Figure 3:**
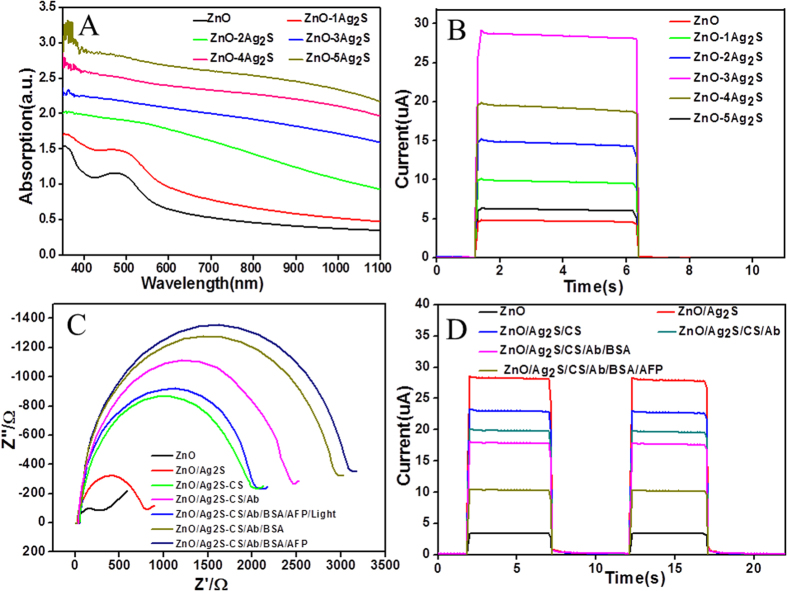
(**A**) Absorption spectra and (**B**) Photocurrent response of ZnO IOs electrode modified with different SILAR cycles of Ag_2_S, (**C**) Electrochemical impedance Nyquist plot and (**D**) photocurrent response of modified ZnO IOs electrodes.

Combining Ag_2_S NPs with ZnO IOs could increase the optical absorption, accelerate charge separation and suppress photo-generated carriers recombination. In order to find the best cycles times of Ag_2_S, electrodes with different cycles of Ag_2_S coatings were studied. Figure 3A shows the UV–vis absorption spectra changes of ZnO/Ag_2_S electrodes with various SILAR cycles. The ZnO IOs demonstrated photonic stop band (PSB) around 490 nm. The PSB in face centered cubic (fcc) photonic crystals could be described by Bragg’s law of diffraction^47^:


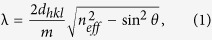


where λ is the central wavelength of PSB, m is the order of the Bragg diffraction, *d*_*hkl*_ is the 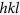 plane distance, *n*_*eff*_ is the average refractive index, and θ is the angle from the incident light to the normal of the substrate surface. For the ZnO IOs, *n*_*eff*_ can be expressed as


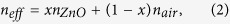


where *x* is the volume ratio of ZnO IOs. Based on Eqs 1 and 2, *x* was deduced to be 0.22 (*n*_ZnO_ = 1.9), which was a little bit smaller than the ideal value (0.26). After the deposition of Ag_2_S, the PSB of ZnO IOs was gradually covered, and the absorption in the visible and near-infrared range gradually increased. It should be noted that with increasing SILAR cycles, the color of the electrode changed from light yellow to brown, which indicated that the amount deposition of Ag_2_S NPs on the ZnO IOs gradually increased, resulting in more light absorption^37^. Figure 3B shows the photocurrent of ZnO/Ag_2_S electrodes with various SILAR cycles. At the beginning, the photocurrent intensity increased with increasing SILAR cycles, and three SILAR cycles of ZnO/Ag_2_S electrodes possessed the optimum, which was attributed to the improved light absorption due to Ag_2_S loading. As the cycle number further increased, photocurrent gradually decreased, because effective surface area with the electrolyte solution decreased due to the excess deposition of Ag_2_S, which blocked the pores of ZnO IOs. Besides, the extra Ag_2_S increased the diffusion resistance to block electron transfer and offered more surface recombination centers^33^,^48^. Thus, three cycle numbers of ZnO/Ag_2_S electrodes were used in the following experience.

### Characterizing the construction process of photoelectrochemical immunosensor

Electrochemical impedance spectroscopy (EIS) was used to analyze the biosensor construction process which is a simple and useful tool for monitoring change of electrode. Figure 3C shows the Nyquist diagrams of electrodes fabricated in each step, with the frequency range 0.1 Hz-100 KHz in a KCl, K_3_[Fe(CN)_6_] and K_4_[Fe(CN)_6_] mixture solution. The electron transfer resistance (Ret) equals semicircle diameter. For the FTO/ZnO electrode, the impedance spectrum was obtained with a very small semicircle, indicating a very small Ret. After Ag_2_S NPs was modified onto the FTO/ZnO electrode, the Ret increased owing to low conductivity of semiconductors. While CS, Ab, BSA and AFP were dropped on the electrodes step by step, Ret increased further correspondingly. This is because of insulating effect of organic molecules, which affects the electronic transfer to electrode surface. EIS indicated that the stepwise fabrication process of immunosensor was successfully designed. It should be noted that the Ret of immunosensor decreases with Xe-lamp illumination due to the improvement in the carrier concentration of the photoelectrodes^49^.

The fabrication of the immunosensor through photocurrent can be also examined. Figure 3D shows photocurrent response of each step modified FTO/ZnO electrodes. Thanks to the sensitization effect of Ag_2_S, the photocurrent significantly increased by 6.2 times than that of FTO/ZnO electrode after the deposition of Ag_2_S NPs on the FTO/ZnO modified electrode^41^. After the successive immobilization of the CS, anti-AFP, BSA and AFP on the FTO/ZnO/Ag_2_S modified electrode, the photocurrent intensity decreased. The fact is that the immobilization of these on the FTO/ZnO/Ag_2_S modified electrodes hindered electronic transmission and increased steric hindrances in electrode/solution interface^50^. Therefore a label-free photoelectrochemical immunosensor was achieved by monitoring the photocurrent change. Figure 1S displays cyclic voltammograms (CVs). When the FTO/ZnO electrode was modified by Ag_2_S NPs, Current response increased over the FTO/ZnO IOs electrode due to the increased surface active sites. Then the CS was dropped on the FTO/ZnO/Ag_2_S electrode, which could form an electron-blocking element and hinder the efficiency electron transfer resulting in current response decrease. After the electrode modification with Ab, BSA and AFP, the current further decreased. CVs also show the successful fabrication process of the electrode.

### Effect of experimental conditions on photocurrent response

**Figure 4:**
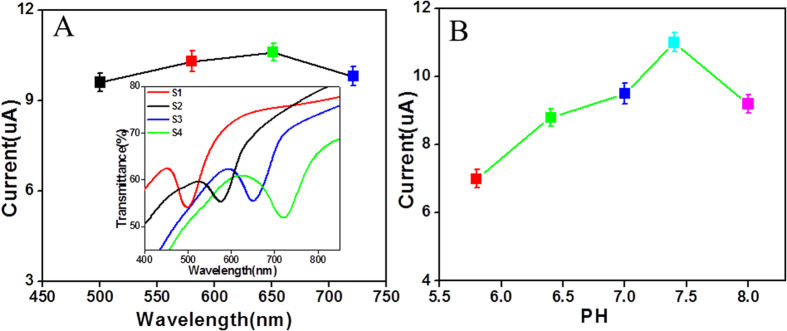
(**A**) The photocurrent of AFP detection (100 ng/ml) to different ZnO IOs electrodes (the inset figure is the transmission spectra of the four samples), (**B**) pH of the detection solution on the photocurrent response of the immunosensor toward 100 ng/ml AFP in 0.1 M PBS.

In order to find an effective macroporous structure, four electrodes with PSB at 500, 580, 652 nm and 721 nm were fabricated. The inset picture of Fig. 4A shows the transmission spectra of the four electrodes, showing the same good structure of IOs sample. The ZnO/Ag_2_S composited electrodes were selected to detect AFP (100 ng/mL). The photocurrent response of composited electrodes to AFP with different PSB positions was shown in Fig. 4A. The photocurrents were 9.6 10.3, 10.6 and 9.8 μA, respectively. The photocurrents had no obvious change with the location of PSB as we previously reported^33^. Considering the stability of the electrode and the activity of Ab, AFP, the PH value in the process of biological detection is also very significant^51^. We tested the photocurrent response of different PH (5.8–8.0) detection solution in order to achieve the optimal effect. As shown in Fig. 4B, the photocurrent obtained at pH = 7.4 was optimal. Therefore, we used detection solution with a pH of 7.4 in the following experiments.

### Photoelectrochemical detection of the immunosensor to AFP

**Figure 5:**
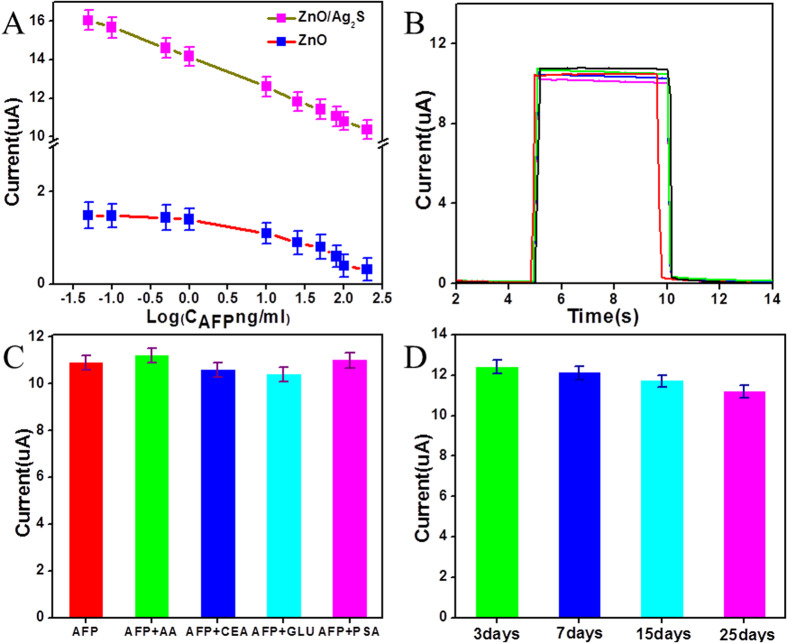
(**A**) The calibration curve of ZnO and ZnO/Ag_2_S composited electrodes for different concentrations of AFP, (**B**) The reproducibility of the immunoassay by detecting 100 ng/mL AFP samples with five electrodes, (**C**) specificity of the immunoassay with 100 ng/mL of AFP without or with 500 ng/ml of (AA), 500 ng/ml of (CEA), 500 ng/ml of (GLU) and 500 ng/ml of (PSA), (**D**) The long-term stability of the immunosensor, detection in PBS solution (0.1 M, PH = 7.4) at potential of 0.6 V.

Photoelectrochemical detection of the immunosensor to AFP was carried out under the optimal immunoassay conditions. Different concentrations AFP (15 uL) were immobilized on optimal electrode after blocking with BSA and the photocurrent responses were obtained. In order to check the influence of Ag_2_S on the sensor performance, immunosensors based on ZnO and ZnO/Ag_2_S composited electrodes were compared. Figure 5A shows the calibration curve of the developed ZnO IOs electrode and ZnO/Ag_2_S composited electrode immunosensor used for the determination of the concentration of AFP. The photocurrent decrement was proportional to the logarithmic value of AFP concentration for ZnO/Ag_2_S electrode. The regression equation was ΔI_1_ = −1.60 logC_AFP_ + 14.09, ranging from 0.05 ng/mL to 200 ng/mL with a correlation coefficient of 0.999 and a low detection limit of 8 pg/ml. Here, ΔI_1_ was the photocurrent of FTO/ZnO/Ag_2_S/CS/anti-AFP/BSA electrode incubated with 15 μL different concentrations of AFP. FTO/ZnO/CS/anti-AFP/BSA electrode was incubated with 15 μL different concentrations of AFP to obtain ΔI_2_ of ZnO IOs electrode, the regression equation was ΔI_2_ = −0.327 logC_AFP_ + 1.38 for immunosensor without Ag_2_S NPs in the range from 0.5 to 50 ng/mL. It is clear that ZnO/Ag_2_S electrode showed a higher photocurrent, better linearity and sensitivity than that of ZnO electrode. The composited electrode has a wider linear range and lower detection limit than that of ZnO electrode, which was significant for the detection of AFP. The results showed that the performance of composite structure immunosensor was acceptable and promising. Compared with previous reports shown in Table 1, the proposed photoelectrochemical immunoassay exhibits enough sensitivity for the detection of AFP. Moreover, it also proved that the proposed lable-free sensitization strategy of the detection biomarkers for early diagnosis and disease surveillance was particularly promising.

### Reproducibility, specificity and stability of the immunosensor

The reproducibility of five immunosensors was evaluated towards to 100 ng/mL of AFP, and the relative standard deviation (RSD) of the five independent assay systems was 2.6%. As shown in Fig. 5B, no obvious changes could be found, showing good precision and acceptable reproducibility.

Specificity is vital to immunoassay, since the nonspecific adsorption can influence the sensitivity. To survey the photocurrent response originated from specific binding, the photocurrent of five electrodes with 100 ng/mL of AFP, without or with 500 ng/ml of ascorbic acid (AA), 500 ng/ml of carcinoembryonic antigen (CEA), 500 ng/ml of glucose (GLU) and 500 ng/ml of prostate specific antigen (PSA) was investigated, as seen in Fig. 5C. No obvious photocurrent change was observed, suggesting that the photocurrent responses arose from the interaction of AFP and anti-AFP, which were specific without much interference from nonspecific adsorption. The immunosensor possessed a satisfactory specificity.

The long-term storage stability of the immunosensor was investigated. When the immunosensor was stored in a refrigerator at 4 °C, photocurrent response was got in the detection of 10 ng/mL AFP after different storage time (3, 7, 15, 25 days), as shown in Fig. 5D. The immunosensor still remained more than 90% photocurrent after 25 days, which showed good long-term storage stability.

## Conclusions

A simple and effective method was proposed to fabricate low-toxicity Ag_2_S NPs modified ZnO electrodes. ZnO IOs composited with Ag_2_S NPs could not only increase the effective utilization and absorption of light but also accelerate the electron transfer and restrain recombination of charge for ZnO/Ag_2_S structure upon irradiation due to the effective matching of energy levels between ZnO and Ag_2_S. The optimal cycle numbers of Ag_2_S deposited on ZnO IOs were studied and the three cycles ZnO/Ag_2_S composited electrode showed higher photocurrent response, wider linear range and lower detection limit. The designed immunosensor based on composited electrode for quantitative detection of AFP exhibited high sensitivity, good reproducibility, and long-term stability. This proposed photoelectrochemical method can be expanded readily for detecting other cancer biomarkers and pathogens.

## Additional Information

**How to cite this article**: Jiang, Y. *et al*. Photoelectrochemical detection of alpha-fetoprotein based on ZnO inverse opals structure electrodes modified by Ag_2_S nanoparticles. *Sci. Rep.* **6**, 38400; doi: 10.1038/srep38400 (2016).

**Publisher's note:** Springer Nature remains neutral with regard to jurisdictional claims in published maps and institutional affiliations.

## Supplementary Material

Supporting Information

## Figures and Tables

**Table 1:** Analytical performance of various methods for AFP immunoassays.

Method	Linear range [ng/mL]	Detection limit [pg/mL]	Ref.
Photoelectrochemical immunosensor	0.05–200	8	This work
Electrochemical immunosensor	0.05–150	20	11
Fluoresencent immunosensor	0.025–5	12	9
Electrochemiluminescence immunosensor	0.5–600	480	8
Photoelectrochemical immunosensor	0.1–500	10	32
