# Supplementary material for: Photoelectrochemical detection of alpha-fetoprotein based on ZnO inverse opals structure electrodes modified by Ag2S nanoparticles
Source: Sci Rep. 2016 Dec 6;6:38400. doi: 10.1038/srep38400 (PMC5138818; doi:10.1038/srep38400)
Supplement: Supporting Information [file srep38400-s1.pdf]

Supplementary Materials for

Photoelectrochemical detection of alpha-fetoprotein based on ZnO inverse opals structure electrodes modified by Ag_2S nanoparticles

Yandong Jiang[a], Dali Liu[a]*, Yudan Yang[b], Ru Xu[a], Tianxiang Zhang[a], Kuang Sheng[a], Hongwei Song[a]*

a. State Key Laboratory on Integrated Optoelectronics, College of Electronic Science and Engineering, Jilin University, 2699 Qianjin Street, Changchun 130012, China

b. China-Japan Union Hospital of Jilin University, Changchun 130012, China

Corresponding to:

Prof. Dali Liu E-Mail: ldl@jlu.edu.cn Fax: 86-0431-85168270

Prof. Hongwei Song E-Mail: songhw@jlu.edu.cn Fax: 86-0431-85155129

1. Experimental Section

1.1 Materials.

Zinc nitrate (Zn $(NO_3)_2 \cdot 6H_2O$), methyl methacrylate (MMA) were purchased from Tianjin Chemical Plant Co., Ltd (Tianjin, China). Citric acid, ethanol and ascorbic acid (AA) were purchased from Beijing Chemical Plant Co. (Beijing, China). Tetraethoxysilane (TEOS) was purchased from Sinopharm Chemical Reagent Co. (China). Chitosan (CS) was purchased from solarbio Co., Ltd (China). Alpha-fetoprotein (AFP), carcinoembryonic antigen (CEA) prostate specific antigen (PSA), glucose (GLU) and Anti-AFP antibody (Ab, polyclonal antibody) were purchased from Beijing Boisynthese Biotechnology Co., Ltd. (Beijing, China). Bovine serum albumin (BSA, 96–99%) was purchased from Beijing DingGuo Biotechnology Company (Beijing, China). Silver nitrate ($AgNO_3$, 99.5%) and sodium sulfide nonahydrate (Na_2S, 98.0%) were purchased from Sigma-Aldrich (Steinheim, Germany). Phosphate buffered saline (PBS) was prepared in a conventional way for configuring the AFP and Ab with pH = 7.2 (0.01 M) and the test solution in 0.1 M PBS solution (PH=7.4), washing buffer was 0.1 M PBS (PH=7.4), blocking buffer was 0.1 M PBS (PH=7.4) containing 1% BSA, respectively. All reagents were of analytical purity and were used without further purification. All aqueous solutions were prepared with deionized water (DI water, 18.25 MΏ/cm), which was obtained from a water purification system.

1.2 Apparatus.

UV - visible (UV-VIS) transmittance spectra was measured using a Shimadzu UV-1800 scanning spectrophotometer ranging of 300-1100 nm. The morphology of the samples was characterized using a JEOL JSM-7500F field emission scanning electron microscope (SEM) (Japan). X-ray diffractometry (XRD) analysis was carried out using a RigakuD/max 2550 X-ray diffractometer. Electrochemical impedance spectroscopies (EIS) and Electrochemical tests were performed with a model CHI660D electrochemical analyzer (ChenHua Instruments Co., Ltd., Shanghai, China)

using a conventional three electrode system, comprising of a platinum wire as counter electrode, a saturated calomel electrode (SCE) as reference electrode, and the modified electrode with a geometrical area of 1.0 $\pm$0.1 cm^2 as working electrode. The cyclic voltammetry curves (CVs) were recorded at scan rate of 100 mV /s. All photocurrent measurement were conducted under a 500 W xenon lamp of irradiation at a constant potential of 0.6 V (relative to a saturated Ag/AgCl electrode) in 0.1 M PBS (pH = 7.4).

1.3 Preparation of Electrode.

1.3.1 Preparation of ZnO IOs.

First, the homogeneously dispersed PMMA microballoon were synthesized according to the previous report [1]. the FTO (sheet resistance 30 Ω/cm^2; Qiseguang Glass Co. Ltd., Dalian) substrate was immersed in the mixed solution of H_2O_2/H_2SO_4 with the volume ratio of 1: 3 and washed with DI water to make the surface hydrophilic. Then the FTO substrate was washed ultrasonically in the solution of sodium hydroxide for 15 min and washing with DI water.

Vertical sedimentary PMMA templates were got by self-assembly method. And the hydrophilic FTO substrate was Placeed in the beaker with the prepared PMMA colloidal suspension and placed in an oven at 32 ℃ for 20 h. Subsequently, PMMA colloidal spheres were slowly self-assembled into a highly ordered array on the FTO substrate by the surface tension produced in the process of the liquid evaporation. Afterwards, the PMMA template was annealed at 120 ℃ for 1h to strengthen its physical property.

$Zn(NO_3)_2.6H_2O$ and citric acid were dissolved in ethanol solution to form the precursor solution. after even agitation the appropriate amount of TEOS was added and stirred until becoming the colorless transparent solution. Next, the prepared precursor solution was infiltrated into the interval of the PMMA templates by capillary force. Finally, the samples were heated to 500 ℃ for 3 hours to burn the

PMMA template so that the ZnO IOs structure was got.

1.3.2 Preparation of ZnO IOs-Ag_2S NPs electrode.

Ag_2S NPs deposition on ZnO IOs via a SILAR method with slight modification [2], Ethanol methanol was used as solvents, Because of their lower surface tension, leading to a superior penetration ability on ZnO IOs. Briefly, the ZnO IOs substrate was first dipped into a 0.1 M $AgNO_3$ ethanol solution for 1 min, rinsed with ethanol, dipped into a 0.1 M Na_2S ethanol/water solution with the volume ratio of 1: 3 for 3 mins and then rinsed with ethanol. The two-step procedure calls one SILAR cycle and the amounts of silver sulfide adsorption would be increased by cycle numbers. This cycle process was repeated 1 to 5 times. Finally, the as-prepared ZnO/Ag_2S composited electrode was dried.

CS solution (0.5 wt %) was prepared by dissolving Chitosan powder in 1% acetic acid. CS solution (10 μL) was dripped onto the FTO/ZnO/Ag_2S electrode and dried at room temperature, and then washed with washing buffer. Then, Ab (50 μg /mL, 15 μL) was conjugated onto the electrode and placed at 4 ℃ 12h to obtain the FTO/ZnO/Ag_2S/CS/Ab electrode. the electrode was washed with washing buffer. To avoid nonspecific adsorption, blocking buffer was added and electrode was put at 37 ℃ for 30 min, then rinsed with washing buffer and restored at 4 ℃ for subsequent photoelectrochemical immunoassays.

1.4 Photoelectrochemical measurements.

Different concentrations of AFP solution (15 μL) were dropped onto the prepared electrode and incubated at 37 ℃ for 1h to get the photoelectrochemical biosensor. Then, the sensor was rinsed carefully with wash buffer to remove the Ab which hadn’t connected to the photoelectrochemical biosensor. Finally, Photocurrent measurement was carried out using a 500 W xenon lamp irradiate at a constant potential of 0.6 V in test solution (0.1M PBS with PH=7.4) at room temperature.

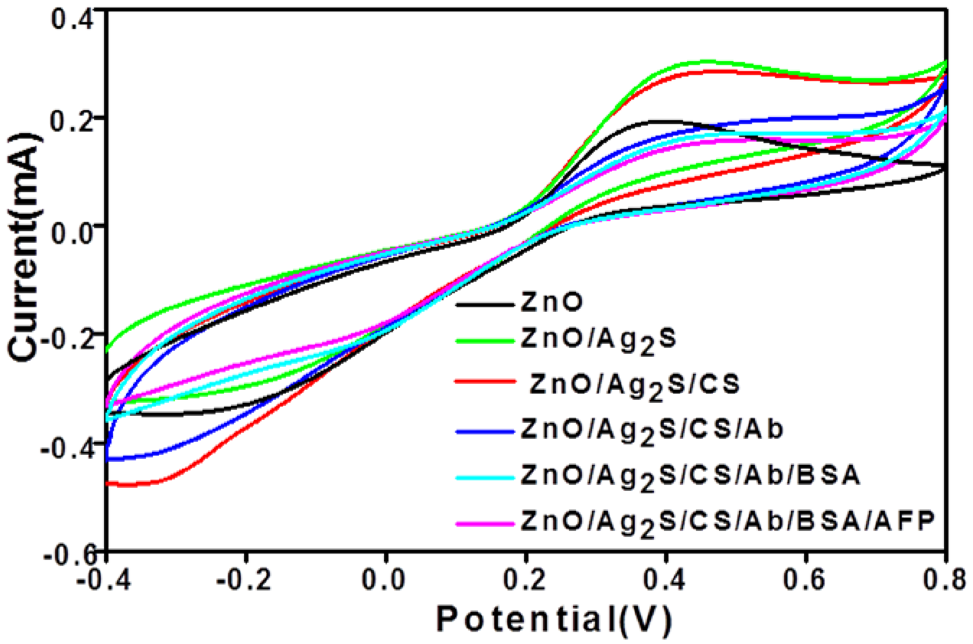

Fig. S1. CVs of the immunosensor fabrication progress in 0.1M PBS (PH=7.4).

Reference

1. Xia, L., Song, J., Xu, R., Liu, D., Dong, B., Xu, L., and Song, H. Zinc oxide inverse opal electrodes modified by glucose oxidase for electrochemical and photoelectrochemical biosensor. Biosensors & Bioelectronics **59**, 350-357 (2014).

2. Tubtimtae, A., Wu, K.-L., Tung, H.-Y., Lee, M.-W., and Wang, G.J. Ag_2S quantum dot-sensitized solar cells. Electrochemistry Communications **12**, 1158-1160 (2010).
